# Modelling microbial communities using biochemical resource allocation analysis

**DOI:** 10.1098/rsif.2019.0474

**Published:** 2019-11-06

**Authors:** Suraj Sharma, Ralf Steuer

**Affiliations:** Humboldt-Universität zu Berlin, Institut für Biologie, FachInstitut für Theoretische Biologie (ITB), Invalidenstr. 110, 10115 Berlin, Germany

**Keywords:** cyanobacteria, trait-based modelling, ecosystems biology, marine ecology, flux-balance analysis

## Abstract

To understand the functioning and dynamics of microbial communities is a fundamental challenge in current biology. To tackle this challenge, the construction of computational models of interacting microbes is an indispensable tool. There is, however, a large chasm between ecologically motivated descriptions of microbial growth used in many current ecosystems simulations, and detailed metabolic pathway and genome-based descriptions developed in the context of systems and synthetic biology. Here, we seek to demonstrate how resource allocation models of microbial growth offer the potential to advance ecosystem simulations and their parametrization. In particular, recent work on quantitative resource allocation allow us to formulate mechanistic models of microbial growth that are physiologically meaningful while remaining computationally tractable. These models go beyond Michaelis–Menten and Monod-type growth models, and are capable of accounting for emergent properties that underlie the remarkable plasticity of microbial growth. We outline the utility and advantages of using biochemical resource allocation models by considering a coarse-grained model of cyanobacterial growth and demonstrate how the model allows us to address specific questions of relevance for the simulation of marine microbial ecosystems, including the physiological acclimation of protein expression to different environments, the description of co-limitation by several nutrients and the differential use of alternative nutrient sources, as well as the description of metabolic diversity based on our increasing knowledge about quantitative cell physiology.

## 1. Introduction

Microbial organisms are integral parts of the Earth’s biogeochemical cycles and play key roles in almost all environments and ecosystems. Microbes typically form complex, interacting, and dynamically changing communities, with examples ranging from marine plankton communities to the human microbiome. To understand the organizing principles and the functioning of such communities is of paramount importance for a vast number of basic and applied research questions, including questions pertinent to biotechnology, climate change and human health [1–4]. Despite the significant advances in our ability to observe and characterize biological systems, however, understanding the interactions and the emergent dynamics of microbial communities remains a fundamental, and truly transdisciplinary, challenge [5–9].

Traditionally, ecosystem dynamics and microbial communities are the realm of microbial ecology, with a long history and a wealth of results concerning the organization, stability and functioning of (microbial) ecosystems [1,10]. In the past century, a variety of modelling approaches have been developed to address fundamental ecological questions, ranging from understanding patterns of biodiversity to predicting the response of ecosystems to changing environmental conditions [5,7,10–12]. In this context, descriptions of microbial growth range from phenomenological ‘black box’ models to more elaborate trait-based models of growth [5,7]. It has been noted, though, that current theoretical approaches to microbial growth are still dominated by the classic Monod or Michaelis–Menten functional form [6,13]. While undoubtedly highly successful, Monod-type models of growth exhibit a number of limitations. For example, it has been argued that the constant parameters used in the Monod equation cannot account for the observed plasticity of microbial physiology [14]. Likewise, it has been emphasized that, despite the significant advances in genome sequencing and quantitative high-throughout methods, the complexity of mechanistic ecosystem models, and in particular the description of microbial growth within these, have not changed substantially since they were developed in the 1970s [13].

Parallel to progress in theoretical and experimental microbial ecology, the past two decades have seen an unprecedented advance in our understanding of microbial molecular physiology—mainly driven by advances in our ability to monitor, measure and modify the inner workings of cells. Theoretical and computational descriptions of microbial metabolism, facilitated by large-scale metabolic network reconstruction and constraint-based analysis, have become established tools in molecular systems biology [15–17]. Curated genome-scale reconstructions (GSRs) of metabolic networks are available for an increasing number of microbial organisms [18–20], and are increasingly recognized as a promising resource for studying metabolic interactions within microbial communities [2–4,8,9,21,22],

More recently, also the quantitative growth physiology of bacteria has gained renewed attention, with numerous studies providing novel insights into the principles of microbial growth and resource allocation [23–27]. Key observations concern the ‘laws’ and trade-offs of microbial growth. In continuation of the classic studies of bacterial growth physiology [23], a number of studies have recently addressed the covariation between the cellular composition and the growth rate of microorganisms [24,25]. Theoretical descriptions of microbial resource allocation include coarse-grained models that describe the fundamental processes of cellular growth by partitioning the proteome into few essential classes [26,28–31], as well as large-scale constraint-based models that take into account the costs and benefits of each individual gene [32–35]. In particular, the concept of resource balance analysis [32,34,36] and related methods, such as metabolism and macromolecular expression models [33], metabolic modelling with enzyme kinetics [37], and conditional flux-balance analysis (FBA) [31], show that quantitative models that predict quantitative protein expression are feasible on the genome-scale—and can be extended to time-varying environments [35,38]. As yet, however, quantitative modelling of microbial resource allocation is mostly restricted to well characterized model organisms in typical laboratory or biotechnological environments.

The purpose of this work is therefore to outline a bridge between these recent studies on microbial resource allocation and current models of microbial ecology. We argue that biochemical resource allocation models offer significant potential to advance ecosystem simulations beyond current applications of constraint-based analysis of microbial metabolism. In particular, we seek to demonstrate that biochemical resource allocation models, as defined below, can be constructed and parametrized for large classes of microbial organisms based on available biochemical and physiological data; and are, unlike Monod-type models, capable of exhibiting emergent properties of growth, such as photoacclimation or a preferential hierarchy of nutrient sources. Our study is motivated by recent calls for a new generation of plankton models to better capture the emergent properties of marine ecosystems [6,13]. As will be demonstrated below, biochemical resource allocation models follow the rationale described by Allen & Polimene [6] to design a generic cell model that captures the essence of key physiological activities and that is based on a robust physiological formulation of competing physiological activities—and therefore is able to reproduce biogeochemical and ecological dynamics as emergent properties.

The paper is organized as follows: in the first section, we briefly recapitulate computational models of microbial growth. In the second section, we provide an overview of metabolic network reconstruction and recent biochemical models of cellular resource allocation. In the following section, we consider a coarse-grained model of phototrophic (cyanobacterial) growth and describe its parametrization. In the subsequent section, we discuss properties of relevance for the simulation of microbial ecosystems, such as metabolic plasticity, cellular growth laws and co-limitation by several nutrients, as well as the representation of microbial diversity and the emergence of a preferred hierarchy of nutrient uptake. In the sixth and seventh sections, we briefly present a case study: the co-existence of two phytoplankton species with a gleaner–opportunist trade-off. In the final section, we provide a discussion and outlook.

## 2. Models of microbial growth

Assuming a chemostat-like setting, the growth dynamics of a population of (genetically homogeneous and well mixed) cells can be described by an ordinary differential equation,

2.1 $$\frac{\text{d}\rho }{\text{d}t}=\mu \cdot \rho -D\cdot \rho ,$$

where *ρ* denotes the concentration of cells (described here in units of cells per volume), *μ* denotes the specific growth rate and *D* is the dilution or death rate. The specific growth rate *μ* is a function of the respective environment, and depends on the concentrations of one or more nutrients. Typically, a limiting nutrient *n* with concentration [*n*] is considered that is supplied with the inflow of fresh medium at a concentration [*n*_*x*_]. The respective nutrient dynamics are then described by

2.2 $$\frac{\text{d}[n]}{\text{d}t}=D\cdot ([n_{x}]-[n])-Y^{-1}\cdot \mu \cdot \rho ,$$

where *Y* denotes the yield coefficient, defined as the number of microbial cells (or units biomass) per unit of nutrient.

To evaluate the dynamics of the system requires knowledge of the specific growth rate *μ* as a function of the concentration of the limiting nutrient *n*. To this end, among the most widely used approaches is to make use of the hyperbolic dependency proposed by Monod in 1949 [39],

2.3 $$\mu ([n])=\frac{\mu^{\max}\cdot [n]}{K_{A}+[n]},$$

where *μ*^max^ denotes the maximum specific growth rate of the microorganism in this environment and *K*_*A*_ denotes the half-saturation constant, i.e. the concentration of the limiting substrate at which the specific growth rate is half the maximal rate. The Monod equation is identical to the Michaelis–Menten equation of enzyme kinetics and represents an empirical description of microbial growth. Its constant parameters are typically estimated for specific environmental conditions and reflect a particular strain or species, and its respective functional traits relate to nutrient uptake and growth [7,14]. Over the past decades, there have been several advances and alternative formulations of growth models, such as the Droop model [40] that introduces internal nutrient quotas. For phototrophic microorganisms, modifications are typically required to account for the effects of photoinhibition—the decrease of the specific growth rate for high light intensities [26]. A widely used equation to describe phototrophic growth in dependence of the light intensity *I* is the Haldane equation [16],

2.4 $$\mu (I)=\frac{\mu^{\max}\cdot I}{K_{A}+I+{\left(I/K_{I}\right)}^{2}},$$

where *K*_*I*_ denotes the impact of photoinhibition. In the absence of photoinhibition (*K*_*I*_ → ∞) the model is identical to the Monod equation with light as the limiting substrate. See, for example, Lee *et al.* [41] for a review on empirical growth models and their parametrization for different microalgae.

Equations of the form (2.1) and (2.2) have been studied extensively [10] and are commonly used in ecosystem simulations [5,10,11,42]. The description can be readily extended to multiple microbial strains with concentrations *ρ*_*i*_ and several nutrients *n*_*k*_. We also note that term ‘nutrient’ refers here to any compound or variable that may affect the specific growth rate, including temperature or modulation of growth by quorum sensing. In this case, knowledge of the functional forms of all specific growth rates *μ*_*i*_ in dependence of the variables is required (typical examples for multi-nutrient rate equations are discussed below in the section ‘Acclimation, trade-offs and co-limitation’).

The use of such multi-variable phenomenological rate equations remains ubiquitous in current models of ecosystems [7,11,13,14,42,43]. It has been emphasized recently [6,13], however, that these empirical growth models do not necessarily reflect our vast recent increase in knowledge about the quantitative physiology of microbial growth. The challenge before us is therefore to combine the conceptual simplicity of empirical growth models with molecular properties of microbial growth.

## 3. Metabolic reconstructions and beyond

Models of microbial growth that incorporate internal structure and aspects of physiology are not new. Examples include the (still empirical) model of Droop [40] as well as other ‘internal-quota’ models—each representing a cell with one or more internal variables, and typically allowing for adjustments in the composition of cellular biomass [5]. Likewise, models that incorporate cost–benefit consideration have been proposed, most notably by JA Raven [44] and RJ Geider [45]. In the following, we build on these ideas and incorporate recent approaches to biochemical models of microbial growth [16,17].

In particular, over the past two decades, GSRs of microbial metabolism have reached maturity and are available for a rapidly increasing number of (sequenced) microbial organisms [18–20]. GSRs provide a comprehensive account of biochemical interconversions between small molecules (metabolites) within a cell or organism. Constraint-based methods, such as FBA, then allow to efficiently interrogate GSRs, and thereby enable accurate estimation of the stoichiometric and energetic synthesis costs of cellular constituents.

The predictive success of FBA is based on the fact that the analysis does not require extensive knowledge about kinetic parameters and regulatory interactions. Rather, the implementation of FBA and related methods are based only on knowledge of the stoichiometric matrix (i.e. the GSRs itself) and typically involve the assumption of evolutionary optimality: unknown regulatory interactions are replaced by the assumption that the resulting flux solutions satisfy a given optimality criterion, such as the maximization of a biomass objective function [15]. Based on the combination of GSRs with linear programming, FBA and related methods have been highly successful to predict maximal growth yields of microbial organisms and other properties of biotechnological relevance [15].

Despite their predictive success, however, the conventional formulation of FBA also exhibits several shortcomings. In particular, traditional analyses of GSRs fail to account for the enzymatic costs required for cellular metabolism, and hence growth. To this end, among other improvements detailed elsewhere [46], a new generation of constraint-based models were recently developed that offer significant potential to advance our understanding of ecosystems.

## 4. Models of cellular resource allocation

Building upon GSRs, a new generation of constraint-based microbial growth models has been developed recently, based on the concept of cellular resource allocation [32–36]. Different from FBA, these models aim to predict protein expression and cell compositions of microbes in specified (albeit, with the exception of [31,35,38], constant) environments. Resource allocation models are based on the insight that the (maximal) flux of an enzyme-catalysed biochemical reaction is typically constrained by the amount of the respective enzyme. Since enzymes are themselves the products of metabolism, incorporating enzyme-dependent flux constraints gives rise to a self-consistent description of microbial growth: for any given growth rate *μ* the set of cellular enzymes must be sufficient to sustain the synthesis of the required precursors to allow for the translation of the set of catalysing enzymes itself, as well as for the synthesis of all other (non-enzymatic) compounds within a cell.

In the following, while many of our arguments hold also for a more general class of resource allocation models [26,28], we will focus on a particular implementation of resource balance models based on linear constraints [31,32,34,35]. These models are formulated as linear programs (LPs) and are based (only) on linear constraints between reaction rates and intracellular macromolecules, and hence can be solved efficiently.

More formally, the models are based on a description of the intracellular protein allocation of growing cells. The concentration of a protein *P*_*k*_ can be described by the equation,

4.1 $$\frac{\text{d}[P_{k}]}{\text{d}t}=\gamma_{k}-\mu \cdot [P_{k}],$$

where *γ*_*k*_ denotes the respective translation rate. In steady state, the translation rate has to match the dilution term *μ* · [*P*_*k*_] of cellular growth (additional protein degradation can be readily included). The sum of all translation rates is constrained by the available ribosomal capacity and hence by the number of ribosomes.

To account for the synthesis of metabolic precursors and other cellular components, the interconversion of internal metabolites *m* is described by a stoichiometric matrix *N* and a vector *ν* that denotes the rates of (spontaneous or enzyme-catalysed) interconversion rates,

4.2 $$\frac{\text{d}[m]}{\text{d}t}=N\cdot \nu -\mu \cdot [m].$$

Typically, intracellular metabolism is assumed to be at steady state and the dilution terms for intracellular metabolites are neglected due to the high turnover rate of metabolites compared to their dilution rate by growth. Hence, the mass-balance constraint on intracellular reaction fluxes simplifies to *N* · *ν* = 0.

To account for biochemical resource allocation, the rates of those reactions that are catalysed by proteins are constrained by the amount of the respective catalysing proteins

4.3 $$\nu_{k}\leq k_{\mathrm{cat},k}\cdot P_{k},$$

where *k*_cat,*k*_ denotes the specific activity of the enzyme or protein. The maximal uptake rate *ν*_*T*_ of an external nutrient *n*_*x*_ can be further constrained by the concentration of the respective nutrient and the amount of the respective transporter complex *P*_*T*_,

4.4 $$\nu_{T}\leq \frac{\left[n_{x}\right]}{K_{M}+[n_{x}]}\cdot k_{\mathrm{cat},T}\cdot P_{T}.$$

The uptake constraints can be modified, for example, to also account for diffusion limitations of nutrient uptake as described by Bonachela *et al.* [14] by adding an additional constraint.

The constraints and equations summarized above, together with the assumption of a constant cell density, provide a quantitative description of microbial growth. To obtain a prediction of the growth rate in a specific environment, the model is solved using the assumption of parsimonious resource allocation. That is, the assumption that metabolic fluxes and the corresponding protein levels are organized such that they give rise to a maximal growth rate in the respective environment (assumption of evolutionary optimality). Hence, similar to FBA [15] and other constraint-based analysis, the assumption of optimality replaces unknown regulatory mechanisms.

The required parameters for model construction are: (i) the metabolic network (as encoded in the stoichiometric matrix *N* and the associated enzyme–reaction relationships). These data are available as part of a metabolic network reconstruction; (ii) the composition of the catalysing enzymes (in terms of amino acids and possible co-factors). For most enzymes this information is readily available and part of reaction databases; as well (iii) as the specific activity *k*_cat_ of each catalysing enzyme and, if required, the half-saturation constants for transporter reactions. While quantitative data are still scarce, in particular for non-model organisms, specific activities for a wide range of enzymes can be sourced from suitable databases, such as BRENDA [47], and are therefore (at least approximately) available. As we have argued previously [31,35], reasonable estimates for all required parameters exist.

We note that the respective models may involve different levels of complexity: the biochemical resource allocation model can be constructed on a genome-scale. That is, the models include all known individual enzymatic reaction steps of the respective organisms, see for example Goelzer *et al.* [34] or Reimers *et al.* [35]. Alternatively, to reduce the computational burden, simplified models can be constructed by defining coarse-grained enzyme complexes that represent classes of reactions or pathways, see for example Rügen *et al.* [31]. The definition of coarse-grained enzyme complexes, however, entails an approximation that has to reflect the specific research question—and general rules are currently not established. For future work, we envision automated stoichiometric reduction algorithms (i.e. the merging of several individual enzymatic steps into coarse-grained metabolic processes) that preserve desired functionalities of the network. Such algorithms were recently developed for genome-scale metabolic reconstructions [48], but have yet to be adapted for resource allocation models.

In the following, irrespective of their size, we refer to the class of models outlined above as biochemical resource allocation models (BRAMs). Computationally, for any given growth rate, the resource allocation model gives rise to a LP and hence can be solved efficiently. The maximal growth rate is then identified using bisection, see Material and methods for computational details.

## 5. A model of phototrophic growth

**Figure 1.**
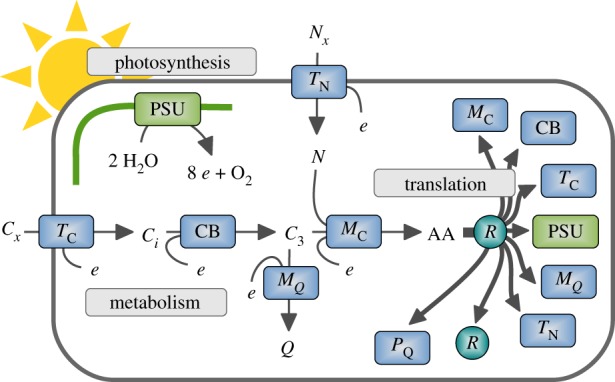
A coarse-grained biochemical resource allocation model of phototropic growth. The model consists of eight protein complexes that catalyse five metabolic and transport reactions, as well as protein translation, light harvesting and photosynthetic electron transport. Extracellular carbon (*C*_*x*_) and nitrogen (*N*_*x*_) are taken up and converted into amino acids (AA). AA are translated into protein complexes by ribosomes (*R*). All reaction rates are constrained by the respective catalysing protein complexes. Abbreviations are: photosynthetic unit (PSU), carbon uptake (*T*_C_), carbon assimilation (CB), nitrogen uptake and metabolism (*T*_N_), central metabolism and amino acid synthesis (*M*_C_), synthesis of other cellular constituents (*M*_*Q*_) and ribosomes (*R*). Abbreviations of metabolites: external inorganic carbon (*C*_*x*_), internal inorganic carbon (*C*_*i*_), assimilated organic carbon and precursor for biosynthesis (*C*_3_), amino acids (AA), external nitrogen (*N*_*x*_), internal nitrogen (*N*), remaining cellular constituents (*Q*), cellular energy unit (*e*). (Online version in colour.)

To exemplify the utility of BRAMs for microbial ecology, we consider the construction and analysis of a coarse-grained model of cyanobacterial growth. Model reduction was based on previous works [26,31,35,49] to reflect the core limitations of phototrophic growth by light, nitrogen and inorganic carbon. The model is depicted in figure 1 and consists of a light harvesting reaction, five metabolic reactions involving six internal metabolites, as well as eight catalysing protein complexes and their respective translation reactions. In brief, inorganic carbon (*C*_*x*_) is taken up using an energy-dependent transporter (*T*_C_). Intracellular inorganic carbon is assimilated into organic carbon (*C*_3_) using inorganic carbon concentrating mechanisms and the Calvin–Benson cycle (CB). The metabolic intermediate *C*_3_ is converted into amino acids (AA) by a coarse-grained metabolism (*M*_C_).

The biosynthesis of amino acids requires a source of nitrogen (*N*) that is taken up from the environment using an energy-dependent transporter and associated nitrogen metabolism (*T*_N_). For amino acid synthesis, we assume a N : C ratio of ∼1/3 (the cellular N : C ratio is lower due to the remaining non-protein biomass component *Q*). Light harvesting and the photosynthetic electron transport chain are represented by a coarse-grained photosynthetic unit (PSU). The PSU protein complex regenerates cellular energy units *e* (combining ATP and the reductant NADPH into a single energy unit). Amino acids are translated into proteins by ribosomes (*R*), which are themselves protein complexes. The fraction of non-enzymatic proteins is represented by a (quota) protein component *P*_*Q*_. The remaining biomass is lumped into a metabolic component *Q* that is synthesized from the cellular precursor *C*_3_ by the protein complex *M*_*Q*_. All proteins complexes represent aggregates of individual proteins. The model assumes a constant cellular density. The specific growth rate does not directly depend on cell size, but cell size may constrain certain parameters, such as the surface to volume ratio. The full set of equations is provided in the Material and methods.

All enzyme-catalysed reactions are constrained by the amount of the respective enzymes. For example, carbon uptake is constrained by the equation

5.1 $$\nu_{TC}\leq \frac{\left[C_{x}\right]}{K_{C}+[C_{x}]}\cdot k_{\mathrm{cat},T_{C}}\cdot [T_{C}],$$

where [*T*_C_] denotes the amount of uptake transporter (in molecules per cell), $k_{\mathrm{cat},T_{C}}$ denotes the specific catalytic activity of the transport and *K*_C_ denotes the half-saturation constant of the uptake complex. We note that equation (5.1) provides an upper limit only, the actual flux can be less (for example by inactivating a fraction of the uptake transporter). Likewise, additional constraints can be included, such as an upper limit on the uptake flux induced by diffusion limitations [14]. Intracellular reactions are similarly constrained by the maximal catalytic capacities of the respective enzymes and their amounts, e.g. for the carbon assimilation reaction

5.2 $$\nu_{\mathrm{CB}}\leq k_{\mathrm{cat},\mathrm{CB}}\cdot [\text{CB}].$$

The model is parametrized using information about the individual enzymatic and biochemical processes. Using data from Faizi *et al.* [26], the effective size of the (coarse-grained) protein complexes can be approximated by the number of enzymes involved in amino acid synthesis multiplied by the average size (in units of amino acids) per enzyme. Catalytic turnover numbers *k*_cat_ are assigned according to typical values for the respective reactions. For example, the rate of translation per ribosome is approximately 20 amino acids per second, the photosynthetic unit (with photosystems II as rate limiting complex) is assumed to give rise to approximately 250 interconversion per second, the $k_{\mathrm{cat},T_{C}}$ of the carbon transporter is set to 20 s^−1^, the catalytic activity of central metabolism (protein complex *M*_C_) is set to *k*_cat,MC_ = 10 s^−1^. Reasonable parameter ranges for many enzymatic processes (for a generic cell) can be obtained, for example, from Milo & Phillips [50]. The full set of parameters used in the following is provided in the Material and methods.

**Figure 2.**
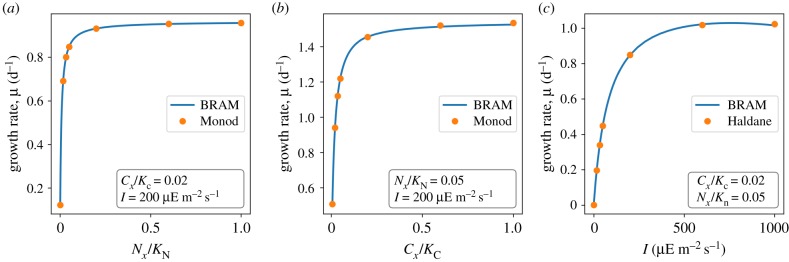
The maximal specific growth rate *μ* as a function of extracellular nutrient concentrations *N*_*x*_ and *C*_*x*_ and light intensities *I*. External nutrient concentrations are reported relative to the half-saturation constant of the respective transporter complex. (*a*) The specific growth rate *μ* as a function of *N*_*x*_/*K*_n_ with constant *I* = 200 μE m^−2^ s^−1^ and *C*_*x*_/*K*_c_ = 0.02. (*b*) The specific growth rate *μ* as a function of *C*_*x*_/*K*_c_ with constant *N*_*x*_/*K*_n_ = 0.05 and light intensity *I* = 200 μE m^−2^ s^−1^. (*c*) The specific growth rate *μ* as a function of light intensity *I* with constant *C*_*x*_/*K*_c_ = 0.02 and *N*_*x*_/*K*_n_ = 0.05. The dots indicate a fit the Monod (for light and inorganic carbon) and Haldane equation (dependence on light intensity), respectively. Abbreviations: *N*_*x*_, external nitrogen concentration; *K*_n_, half-saturation constant of nitrogen transporter; *C*_*x*_, external inorganic carbon concentration; and *K*_c_, half-saturation constant of carbon transporter. (Online version in colour.)

Given the stoichiometric constraints and the assigned parameters, the model gives rise to a computational optimization problem. The optimization problem can be solved as a series of LPs to identify the maximal specific growth rate *μ* in dependence of the availability of extracellular nitrogen and carbon and light intensity *I* (assumption of evolutionary optimality). Figure 2 shows the resulting growth curves as a function of environmental parameters. Similar to previous models [26], the resulting growth curves with respect to external nitrogen (*N*_*x*_) and carbon (*C*_*x*_) concentrations are consistent with Monod kinetics, the dependence of the specific growth rate on the light intensity is consistent with the Haldane equation. Figure 2 shows the respective comparison of the growth rate with the phenomenological rate equations (2.3) and (2.4).

We note that the growth curves shown in figure 2 are emergent properties of the underlying constraints and parameters, i.e. they result as a consequence of the constraints and parameters that characterize the individual biochemical process. While the dependencies on single nutrients are (in this case) similar to the respective phenomenological rate equations, changes in model constraints and parameters entail (sometimes complex) changes in overall growth properties. For example, the apparent half-saturation constants of the organismal growth curves are markedly different from the half-saturation constants of the respective transporter complexes, due to the fact that cells can acclimate to low nutrient conditions by changing the expression of the respective protein complex. More importantly, biochemical resource allocation models allow us to further address trade-offs in protein allocation, limitations by multiple nutrients, including hierarchies of nutrient uptake, as well as other properties relevant for computational models in microbial ecology.

## 6. Acclimation, trade-offs and co-limitation

Biochemical resource allocation models go beyond phenomenological rate equations, and provide insights into acclimation, co-limitation and cellular trade-offs. In particular, concomitant to the overall cellular growth rate, we obtain the distribution of protein resources within the cell as a function of the environmental parameters.

**Figure 3.**
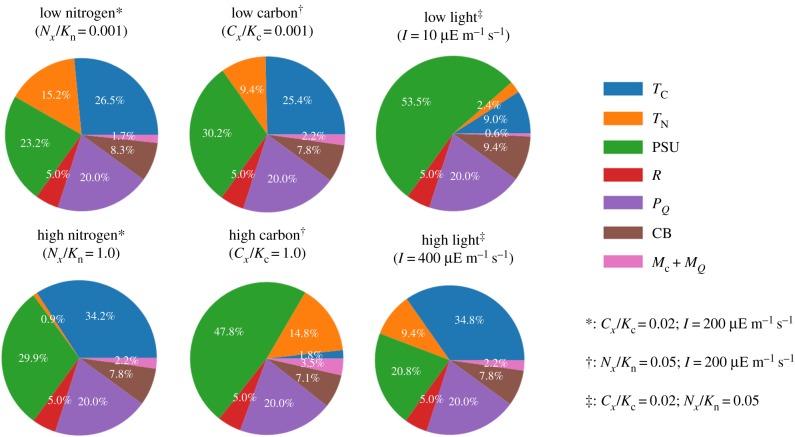
Cellular protein allocation in dependence of environmental conditions. Shown are the relative abundances of ribosomes and coarse-grained protein complexes (relative to total protein) under different growth conditions. The superscripts (*, † and ‡) indicate the parameter values used to specify environmental conditions. (Online version in colour.)

**Figure 4.**
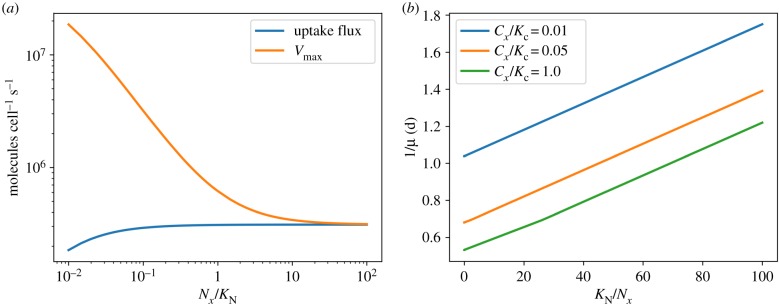
(*a*) The maximal uptake capacity $V_{\max}=k_{\mathrm{cat},T_{N}}\cdot [T_{N}]$ of the nitrogen transport complex versus the actual uptake flux as a function of extracellular nitrogen. In contrast to a description of growth by the Monod equation, more cellular resources are invested into the uptake capacity when nutrients are scarce. (*b*) A Lineweaver–Burk plot of the (inverse of the) growth rate versus the (inverse of the) relative substrate concentration, *K*_N_/*N*_*x*_, for different values of external inorganic carbon. Parallel lines in a Lineweaver–Burk plot correspond to uncompetitive inhibition, whereas a multiplicative dependence of the growth rate on its substrates would result in lines with a identical *x*-intercept. (Online version in colour.)

Figure 3 and table 1 show the relative protein fractions invested into the different biochemical processes in dependence on the environmental conditions. The resource allocation framework allows cells to acclimate to the respective environmental condition and to invest cellular resources into processes that would otherwise limit growth. For example, the maximal uptake rate of the nitrogen transporter complex ($V_{\max}=k_{\mathrm{cat},T_{N}}\cdot [T_{N}]$) and hence the affinity *A* = *V*_max_/*K*_N_ for the extracellular nitrogen source is not constant, but increases with decreasing concentrations of the extracellular nitrogen source. Figure 4*a* shows the maximal uptake rate *V*_max_, as well as the actual nitrogen uptake flux, as a function of available extracellular nitrogen. Similar to the analysis by Bonachela *et al.* [14], and unlike descriptions using the Monod equation, the model accounts for the acclimation of the cell to low nutrient availability—with important consequences for the estimation of phytoplankton abundances in global ocean models. Likewise, protein investments in light harvesting strongly depend on the light intensity, at the expense of investments in other metabolic processes (figure 3 and table 1).

**Table 1.** Cellular protein allocation in dependence of environmental conditions. Values denote the relative abundance (% relative to total proteome) of protein complexes under low and high nutrient conditions. The abbreviations are as in figure 1.

	low			high		
protein	nitrogen	carbon	light	nitrogen	carbon	light
*T*_C_	26.5	25.4	9.0	34.2	1.8	34.8
*T*_N_	15.2	9.4	2.4	0.9	14.8	9.4
PSU	23.2	30.2	53.5	29.9	47.8	20.8
*R*	5.0	5.0	5.0	5.0	5.0	5.0
*P*_*Q*_	20.0	20.0	20.0	20.0	20.0	20.0
CB	8.3	7.8	9.4	7.8	7.1	7.8
*M*_C_ + *M*_*Q*_	1.7	2.2	0.6	2.2	3.5	2.2

Importantly, several recent experimental studies on cyanobacterial growth physiology are in good agreement with the predictions of biochemical resource allocation model [27,51], with changes in protein allocation *in vivo* approximately matching *in silico* predictions. We therefore consider the predictions from biochemical resource allocation models to be a reasonable starting point also for ecological simulations.

Another challenge for phenomenological growth models is to describe the dependence of growth on several potentially limiting nutrients, see Saito *et al.* [52] for a discussion on the concept and types of co-limitation. The most common approaches to implement multiple limitation scenarios rely on either Liebig’s law of the minimum,

6.1 $$\mu =\min\left(\frac{\mu_{1}^{\max}[n_{1}]}{K_{m1}+[n_{1}]},\frac{\mu_{2}^{\max}[n_{2}]}{K_{m2}+[n_{2}]}\right),$$

or the multiplicative form

6.2 $$\mu =\mu^{\max}\cdot \frac{\left[n_{1}\right]}{K_{m1}+[n_{1}]}\cdot \frac{\left[n_{2}\right]}{K_{m2}+[n_{2}]},$$

where [*n*_1_] and [*n*_2_] denote the concentrations of two potentially limiting nutrients and *K*_*m*1_ *K*_*m*2_ the respective half-saturation constants, respectively. As discussed by Saito *et al.* [52] both functional forms are not without problems and there is no clear empirical evidence to assess the merits of either representation. Given its simplicity, the multiplicative form is commonly employed in multi-nutrient models [11,42].

For biochemical resource allocation models the description of growth limitations as a function of two or more nutrients emerges without further assumptions about the functional form of growth equations. As expected, the coarse-grained model described above is not consistent with Liebig’s law of the minimum: growth on a single nutrient, as shown in figure 2, does not exhibit any hard threshold. The absence of such a threshold is due to the fact that, for scarce nutrients, resources are increasingly invested into the respective uptake reactions—hence the limitation between two limiting nutrients is not independent.

More importantly, however, the interdependence of growth limitations by two nutrients implies that the emergent growth curve is also not consistent with the multiplicative functional form: figure 4*b* shows a Lineweaver–Burk plot of the growth rate as a function of nitrogen availability for different values of the external carbon concentration. Parallel lines in a Lineweaver–Burk plot correspond to non-competitive inhibition, whereas the multiplicative functional form of equation (6.2) would result in lines with an identical *x*-intercept. Hence, the absence of carbon acts analogous to uncompetitive inhibition, and affects both the apparent organismal half-saturation constant of growth as well as the maximal growth rate of the cell—again with important consequences when modelling, for example, growth limitations and nutrient dynamics in coupled ecosystem models. While we are not aware of dedicated growth studies to quantitatively investigate the functional dependencies of co-limitation in cyanobacteria, the plasticity of metabolism and re-allocation of proteins makes the strict multiplicative dependence of equation (6.2), as assumed in most current models, highly implausible.

## 7. Metabolic diversity and the cost of regulation

Recent studies have emphasized the representation of microbial community diversity as a fundamental requirement of model ecosystems [5]. Several principles and mechanisms can be used to describe microbial diversity in biochemical resource allocation models. A shown above, cells may acclimate to different environmental conditions, resulting in an inhomogeneous population. If required, the possibility for physiological acclimation can also be restricted within simulations. For example, depending on its evolutionary history, a strain might not be able to access the full range of possible proteins allocated (such a restriction would, for example, be expected for cyanobacterial *Prochlorococcus* strains). The model definition may account for this fact by allowing only limited concentration ranges for certain intracellular protein complexes.

Beyond different acclimation states, however, cellular diversity also arises due to genetically encoded differences between organisms. Firstly, microbial organisms exhibit metabolic diversity with respect to the encoded metabolic functionality within their genomes. As shown by recent studies of the cyanobacterial pan-genome and pan-metabolism [53–55], genome sizes differ significantly—reflecting the different adaptations and lifestyles of organisms. Differences in the set of encoded proteins correspond to different metabolic strategies that are accessible to an organism. For example the accessible modes of energy generation depends on the genetic repertoire of strains [56]. Likewise the accessibility of different nutrient sources may be restricted by the lack of the respective transporter proteins. Secondly, diversity may arise due to differences in enzyme-kinetic parameters. The evolution of enzyme-kinetic parameters is constrained by physico-chemical limits that result in trade-offs between parameters. The protein complex ribulose-1,5-bisphosphate carboxylase/oxygenase (RuBisCO) is a prominent example [57]. As will be shown below, such differences and trade-offs between parameter values may give rise to different cellular growth curves.

To illustrate how BRAMs may represent such genetic diversity, and to illustrate the functional consequences of different metabolic strategies in model simulations, we consider the uptake of two alternative sources of extracellular nitrogen. We assume that, in addition to the nitrogen source *N*_*x*_ considered above, there is a second source of extracellular nitrogen *N*_*y*_ that is permanently available in the environment (analogous to, e.g. atmospheric dinitrogen). The uptake of the permanently available nitrogen source *N*_*y*_ and its conversion to the intracellular nitrogen precurser *N* is facilitated by a set of proteins that is represented in our models by a coarse-grained protein complex *T*_Y_. The synthesis of the protein complex *T*_Y_, however, requires more amino acids and its catalytic turnover number is lower, as compared to the complex *T*_N_. Within their genome, the cyanobacterial strains may either encode one of the two (coarse-grained) uptake protein complexes, *T*_N_ or *T*_Y_, or both. The respective strains are denoted as (*T*_N_)-strain, (*T*_Y_)-strain and (*T*_N_ + *T*_Y_)-strain. The inclusion of both protein complexes within a single genome entails additional cellular costs: a larger genome corresponds to a (slightly) higher fraction of the non-protein biomass *Q*. In addition, further protein machinery is required to facilitate cellular decisions that control the expression of both enzyme complexes. The increased protein machinery is represented by an increased fraction of non-catalysing (quota) proteins *P*_Q_. The exemplary parametrization of the three strains is provided in the Material and methods.

**Figure 5.**
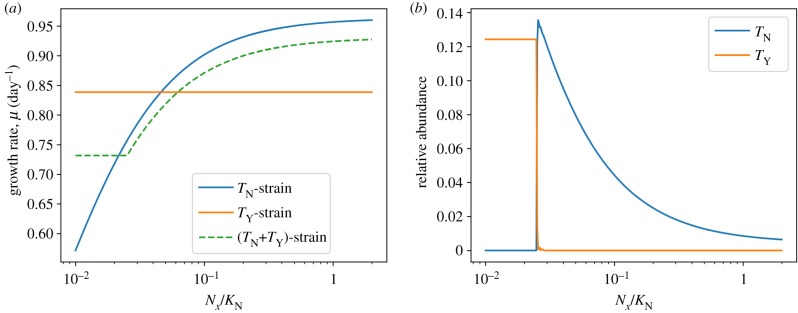
(*a*) The predicted specific growth rate of three different cyanobacterial strains for different concentrations of the external nitrogen source *N*_*x*_ and a basal supply of the alternative nitrogen source *N*_*y*_. The strains are denoted as (*T*_N_)-strain, (*T*_Y_)-strain and (*T*_N_ + *T*_Y_)-strain, and encode either a single uptake mechanism (*T*_N_ or *T*_Y_) or both within their genomes. The (*T*_N_ + *T*_Y_)-strain has a higher biosynthesis cost in terms of increased genome size and additional regulatory proteins and hence exhibits a reduced specific growth rate compared to the streamlined strains. (*b*) Relative abundance (with respect to total proteome) of the nitrogen uptake mechanisms *T*_N_ and *T*_Y_ for the (*T*_N_ + *T*_Y_)-strain. The expression of the respective proteins undergoes a switch between the expression of *T*_N_ and *T*_Y_ in dependence of the availability of *N*_*x*_. (Online version in colour.)

Figure 5*a* shows the growth curves of all three strains as a function of *N*_*x*_ in the presence of a constant basal availability of *N*_*y*_ (figure 5*a*). Figure 5*b* shows the expression of the respective uptake complexes for the (*T*_N_ + *T*_Y_)-strain harbouring both uptake complexes. As expected, the (*T*_Y_)-strain exhibits a constant growth rate, due to the constant basal availability of *N*_Y_. The (*T*_N_)-strain exhibits a Monod-type dependence on the availability of *N*_*x*_, as already shown in figure 2. The combined (*T*_N_ + *T*_Y_)-strain, however, exhibits a switch between two growth regimes: for low availability of external *N*_*x*_, the strain expresses the protein complex *T*_Y_ and uses the nitrogen source *N*_Y_. In this regime, the (constant) specific growth rate is slightly below the rate observed for the (*T*_Y_)-strain due to the increased burden of non-catalytic biomass. If the availability of *N*_*x*_ exceeds a certain threshold, the (*T*_N_ + *T*_Y_)-strain switches its preferred nitrogen source and expresses the protein complex *T*_N_. The growth rate then increases with increasing availability of *N*_*x*_, but always remains below the growth rate of the (*T*_N_)-strain (again due to the increased burden of non-catalytic biomass). Hence, we expect that the (*T*_N_ + *T*_Y_)-strain will be outcompeted in any constant environment, but will have a competitive advantage in (some) environments with variable nitrogen availability.

For our purposes, the example serves to illustrate the following points: (i) biochemical resource allocation models allow us to represent genetic diversity within strains, including differences in genome size and genomes that encode several potential metabolic strategies; (ii) the associated costs of larger genomes, including the costs for additional expression of regulatory proteins can be incorporated into the parametrization of the model (see also Lynch & Marinov [58] for a discussion on the bioenergetic costs of a gene); (iii) the optimal metabolic strategy for any given environment does not have to be specified in advance but is an emergent outcome of model simulation. Strains may switch between different strategies depending on the environment—with important implications for ecosystem models; (iv) within simulations, cells typically exhibit a hierarchy of preferred nutrients. That is, if two nutrient sources are available in the environment, these might either be sequentially consumed (with a preferred nutrient first) or they are simultaneously consumed. Such behaviour is non-trivial to represent within phenomenological Monod-type growth models but emerges naturally for resource allocation models, as recently shown by Wortel *et al.* [59] and Müller *et al.* [60]. A recent study highlights this fact [61] and argues that arguments based on optimal protein allocation indeed enable prediction of microbial nutrient uptake hierarchies in good agreement with experimental observations. The fact that BRAMs can represent and predict such hierarchies in nutrient uptake has again implications for the simulation of ecosystem models.

## 8. A case study: seasonal variation and co-existence

To exemplify the use of BRAMs within ecosystem simulations, we consider a model of phytoplankton diversity recently proposed by Tsakalakis *et al.* [42]. In the following, we do not aim to recapitulate the full study of Tsakalakis *et al.* [42], but focus only on the competition outcomes between different strains of phytoplankton in a constant versus a time-varying environment.

As shown above, the growth physiology of BRAMs is an emergent property of the underlying biochemical parameters. We therefore assume that the biochemical parameters of carbon uptake, as well as nitrogen uptake and metabolism, differ between strains—reflecting microbial diversity of strains. As noted above, our premise is that enzyme-kinetic parameters are subject to physico-chemical trade-offs, for example trade-offs between the half-saturation constant and the maximal catalytic rate of an enzyme. We emphasize that such trade-offs are not an outcome of our modelling approach but need to be specified independently, for example based on detailed biochemical surveys and analysis [57].

**Figure 6.**
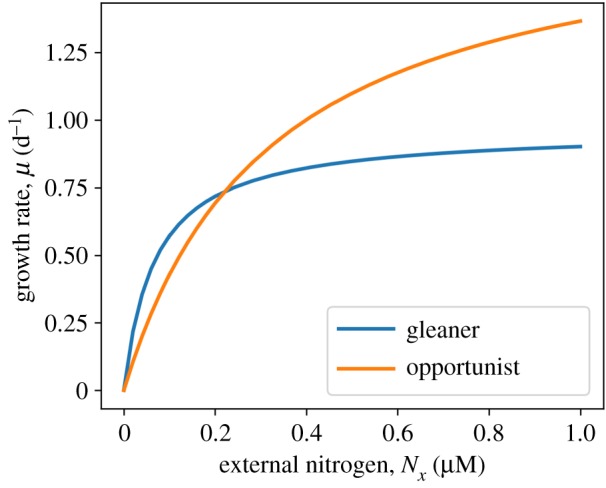
The growth curves of two competing strains of phytoplankton, opportunists and gleaners, in dependence of external nitrogen availability *N*_*x*_. The strains differ in the enzyme-kinetic parameters of their constituent enzyme complexes. Gleaners (K-strategists) have a growth advantage during phases of low external nitrogen availability, whereas opportunists (r-strategists) have a growth advantage at high concentrations of external nitrogen. (Online version in colour.)

In the following, we consider two strains of cyanobacteria (or other phytoplankton). The differences between both strains reflect trade-offs between the maximal specific catalytic activities and the substrate affinities in the protein complexes involved in carbon and nitrogen uptake. The parametrization of both strains is provided in Material and methods and table 2. Differences in parameters give rise to two functional groups of phytoplankton, gleaners (K-strategists) and opportunists (r-strategists). The respective growth curves are shown in figure 6. Gleaners are characterized by a higher affinity towards extracellular nitrogen, and an overall lower maximal growth rate. Opportunists are characterized by a high overall specific growth rate, but a lower affinity for extracellular nitrogen.

**Table 2.** Parameters of the model. The parameter values follow the data used in Faizi *et al.* [26]. If no data were available in the literature, the remaining parameters are estimated${\;}^{⋄}$ based on generic values. The column for ‘Gleaner’ represents the default values.

symbol	definition	gleaner/default	opportunist	source
*V*_cell_	cell volume (μm^3^)	1.8	1.8	[62]
*D*	rate of dilution (d^−1^)	0.25	0.25	[63]
*d*	average cell density (aa cell^−1^)	1.4 × 10^10^	1.4 × 10^10^	[26]
*k*_d_	rate of photo damage	0.56	0.56	⋄
*σ*	effective absorption (m^2^ μmol PSU^−1^)	0.2	0.2	⋄
*n*_PSU_	size of photosynthetic unit PSU (aa molec^−1^)	95 451	95 451	[26]
$n_{T_{C}}$	size of carbon transporter *T*_C_ (aa molec^−1^)	1681	1681	[26]
*n*_CB_	size of Calvin–Benson (CB) proteins (aa molec^−1^)	2000	2000	⋄
*n*_c_	size of carbon metabolism (*M*_C_) proteins (aa molec^−1^)	20 000	20 000	⋄
$n_{T_{N}}$	size of nitrogen transporter *T*_N_ (aa molec^−1^)	10 000	10 000	⋄
*n*_P_	size of protein P (aa molec^−1^)	1000	1000	⋄
*n*_q_	size of metabolism protein *M*_Q_ (aa molec^−1^)	20 000	20 000	⋄
*n*_R_	size of ribosome R (aa molec^−1^)	7358	7358	[26]
*γ*_max_	maximal translation rate (aa s^−1^ molec^−1^)	22	22	[64]
*K*_C_	half-saturation constant of *T*_C_ (μM)	15	15	[65]
*K*_N_	half-saturation constant of *T*_N_ (μM)	10	50	⋄
*k*_cat,PSU_	catalytic activity of PSU (s^−1^)	250	250	[50]
$k_{\mathrm{cat},T_{C}}$	catalytic activity of *T*_C_ (s^−1^)	20	200	⋄
*k*_cat,CB_	catalytic activity of CB (s^−1^)	1	1	⋄
$k_{\mathrm{cat},M_{C}}$	catalytic activity of *M*_C_ (s^−1^)	10	10	[50]
$k_{\mathrm{cat},T_{N}}$	catalytic activity of *T*_N_ (s^−1^)	50	200	⋄
*k*_cat,P_	catalytic activity of *P*_Q_ (s^−1^)	100	100	⋄
*k*_cat,Q_	catalytic activity of *M*_Q_ (s^−1^)	100	100	⋄
*Q*	relative abundance of *Q* w.r.t. biomass	0.5	0.5	⋄
*P*_Q_	relative abundance of *P*_Q_ w.r.t. total proteome	0.2	0.2	⋄

Following Tsakalakis *et al.* [42], we simulate the growth of both strains in two different environments: a constant light environment (control) and an environment with seasonal variations in average light intensity. Extracellular inorganic carbon is assumed to be constant, a (single) source of extracellular nitrogen is supplied via a constant influx. The dynamics of the abundances of gleaners (*ρ*_G_) and opportunists (*ρ*_O_) are described by the following ODEs

8.1 $$\begin{array}{rl} \frac{\text{d}\rho_{G}}{\text{d}t} & =\mu_{G}\cdot \rho_{G}-D\cdot \rho_{G}\\ \frac{\text{d}\rho_{O}}{\text{d}t} & =\mu_{O}\cdot \rho_{O}-D\cdot \rho_{O}, \end{array}\}$$

and the dynamics of external nitrogen is described by

**Figure 7.**
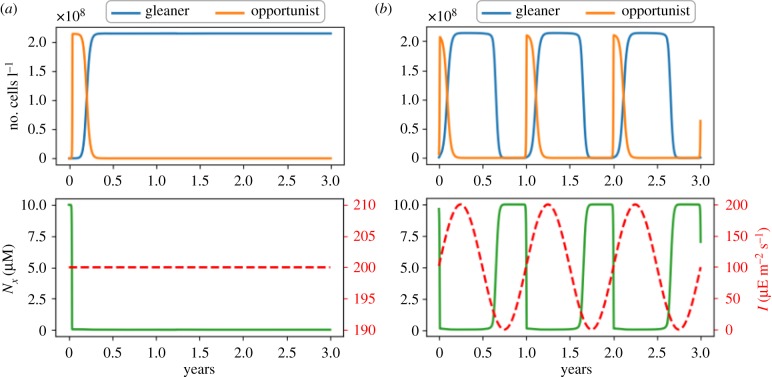
Population dynamics of two competing strains, opportunists and gleaners, under different nutrient and light conditions. (*a*) The gleaner strain outcompetes the opportunist strain under constant light conditions (*I* = 200 μE m^−2^ s^−1^). The lower panel shows the light intensity (red dashed line) and the concentration of available external nitrogen (green solid line). (*b*) Co-existence of both strains under seasonal changes in the average light intensity. The lower panel shows the light intensity (red dashed line) and the concentration of available external nitrogen (green solid line). All simulations are performed using the parameters given in table 2. (Online version in colour.)

8.2 $$\frac{\text{d}[N_{x}]}{\text{d}t}=V_{N}-D\cdot [N_{x}]-\nu_{n,O}\cdot \rho_{O}-\nu_{n,G}\cdot \rho_{G},$$

where *V*_N_ denotes a constant influx, and *ν*_*n*,O_ and *ν*_*n*,G_ denote the specific cellular uptake rates (as emergent properties of the respective models) of external nitrogen by the gleaners and opportunists, respectively. The population dynamics of both strains in constant and time-varying environments are shown in figure 7. Simulations were performed using a Python ODE solver, the growth models are implemented a (series of) LP problems and solved at each time step. The procedure is computationally similar to dynamic FBA (dFBA), an established method for constraint-based analysis [66]. See Material and methods for details.

As shown in figure 7, gleaners outcompete opportunists in a constant light environment, consistent with the competitive exclusion principle. Seasonally changing light intensities, however, induce changes in strain abundances, and hence nitrogen availability. Temporal changes in nitrogen availability then result in the co-existence of both strains. During periods of low light availability, overall strain abundance decreases and the availability of extracellular nitrogen increases. With increasing light intensities, opportunists have a competitive advantage and quickly increase in abundance, thereby decreasing the availability of extracellular nitrogen and shifting the competitive advantage to gleaners until a decreasing light intensity restart the cycle. The simulation results are consistent the corresponding simulations of Tsakalakis *et al.* [42] and demonstrate the feasibility of using biochemical resource allocation models to generate diverse microbial populations for ecosystem simulations.

## 9. Discussion and outlook

As noted by Follows & Dutkiewicz [5], there is currently a vast chasm between the ecologically and biogeochemically oriented parametrizations of growth used in ecological modelling and the metabolic-pathway perspective of microbial growth originating from systems biology and modern genomics. The purpose of this study was therefore to outline the manifold connections between both fields and to show how recent biochemical models of microbial growth may contribute to close the chasm. To this end, we described recent computational models of microbial growth that are based on a description of cellular resource allocation [17,26,30,32,35]. These models allow us to obtain a quantitative account of protein expression that constrain biochemical processes and growth.

The aim of our study was to heed the call of Allen & Polimene [6] to provide growth models based on a robust physiological formulation that allow for trade-offs between resource allocation of competing physiological activities. We propose that biochemical resource allocation models, such as the ones described here, fulfil this paradigm towards a new generation of plankton models. While mechanistic growth models [67], resource-allocation and cost–benefit analysis [5,7,44,45], as well as models based on optimality [68,69], are well established in ecological modelling, biochemical resource allocation models directly build upon metabolic network reconstruction and constraint-based analysis—and therefore reflect the advances in quantitative growth physiology. The predictions from biochemical resource allocation models are often in excellent agreement with detailed physiological and biochemical studies of model strains [27,34,51]. The premise of our study is therefore that these model are a suitable and reasonable starting point for the description of microbial growth also within ecosystems simulations.

Biochemical resource allocation models can be formulated for almost all microbial strains for which a reference genome is available. Supported by recent analysis of the cyanobacterial pan-genome [53–55], and the diversity of energy metabolism in microbes [56], we hypothesize that such models will follow a modular paradigm: there is only a limited number of fundamentally different metabolic strategies available and microbial organisms are a mix-and-match conglomerate of these strategies (with many combinations excluded for biophysical or energetic reasons). The enormous diversity of microbial metabolism then arises from further variations and adaptations of biochemical parameters (including physico-chemical trade-offs), as well as from differences in cellular resource allocation. For example, recent studies indicate that the observed significant differences in the maximal specific growth rates between genetically similar cyanobacterial strains are linked to differences in resource allocation strategies (such as the amount of storage compounds or differences in the PSII/PSI ratio), see Zavřel *et al.* [27] for a brief discussion.

In this study, our aim was not a comprehensive review of computational resource allocation models, but rather to highlight the specific aspects and properties that are of particular importance in simulations of ecosystems, specifically acclimation to different growth environments, co-limitation, and the representation of metabolic diversity. In this respect, the merits of biochemical resource allocation models are as follows:

— BRAMs can be formulated using different levels of complexity, ranging from GSRs that take into account the comprehensive set of cellular proteins and enzymes [34,35], to intermediate representations [31] and smaller coarse-grained models that describe protein complexes that correspond to (agglomerated) cellular processes (an example of the latter was provided in this work). While systematic rules for model reduction are still lacking, we envision algorithmic approaches, similar to the method of Erdrich *et al.* [48], that facilitate the construction of meaningful coarse-grained models is a semi-automated way.
— Model parametrization can be based on available biochemical knowledge provided in databases, such as BRENDA [47], as well as on our increasing knowledge about quantitative cell physiology [50]. The models therefore provide a link between the physico-chemical constraints of enzyme-kinetic parameters and observed growth kinetics. Key quantities for model parametrization are enzyme costs (in terms of amino acids and cofactor requirements) and enzymatic catalytic activities. Information about regulatory mechanisms is not required, but may be added as an additional constraints.
— BRAMs allow us to represent metabolic diversity by taking distributions of parameters (and possible physico-chemical trade-offs between them) into account. Biochemical resource allocation models therefore enable implementation of selection-based approaches—following the Baas-Becking paradigm ‘everything is everywhere but environment selects’ (cited after Follows & Dutkiewicz [5]).
— The analysis of biochemical resource allocation models reveals complex metabolic behaviour, such as switches between different metabolic strategies. Most microbes are capable of more than one metabolic strategy and phenomenological Monod-type models face difficulties to describe transitions between different metabolic strategies. For biochemical resource allocation models the modes of energy generation or nutrient uptake strategies (and their respective hierarchies) emerge as part of the optimization procedure without further specification.
— The latter fact also enables consideration of resource allocation models from an evolutionary perspective to explain how different metabolic strategies and strains with different genome sizes emerge and coexist. Likewise, while the trade-offs affecting individual biochemical parameters are not an outcome of the analysis, the models can provide insights into what factors shape the trade-offs in kinetic parameters within their physico-chemical limits, for example under which environmental conditions would a faster catalytic rate of an enzyme outweigh a reduced specificity?
— Biochemical resource allocation models of the mathematical form discussed here only require linear optimization and hence are computationally tractable. While it is (currently) not possible to formulate kinetic models at the genome-scale, the implementation of biochemical resource allocation models is computationally feasible even for large models [34,35]. Coarse-grained models, such as the one discussed above, can typically be solved fast and efficiently and hence are suitable for ecosystems simulations. In case computational capacity is limiting, it is possible to devise approximate computational schemes (such as lookup tables and interpolation). We note, however, that not all possible constraints may be formulated as a LP problem.

Despite their merits, however, current biochemical resource allocation models are not (yet) the panacea for ecological simulations. We expect that diverse approaches are still needed, along with further improvements of biochemical resource allocation models and other whole-cell systems biology models. In particular, current biochemical resource allocation models may be extended and improved along the following lines: (i) current simulations typically focus on steady state analysis. While it has been shown that biochemical resource allocation models can be solved for time-varying environments [31,35], the computational burden is still significant. It is also of paramount importance to be able to represent phenomena such as storage, bet-hedging or luxury uptake of scarce nutrients (i.e, the uptake of nutrient beyond what is currently required in anticipation of possible future limitations) for ecosystems simulations. These phenomena are, in principle, aspects of resource allocation strategies and hence can be represented by appropriate models; (ii) currently models are based on a metabolic perspective of growth. However, also trade-offs between growth and other cellular properties can be considered, such as the resilience against stress or predation (and the energetic expenses associated with it). Likewise, effects of parameters like temperature and pH currently cannot be adequately described by resource allocation models; (iii) a better understanding of physico-chemical trade-offs in enzyme-kinetic parameters is required, as well as further quantitative growth studies, similar to Zavřel *et al.* [27]. In particular, as yet, laboratory studies concerning resource allocation are often motivated by biotechnological applications and typically do not consider limitations by nutrients other than (inorganic) carbon and light—whereas applications in ecology require that the focus be shifted on limitations typically encountered in natural environments.

Overall, we are confident that biochemical resource allocation models, whose construction is based on reference genomes and increasingly automated [36], offer significant potential for the the computational study of ecosystems and microbial communities—and go beyond current growth models based either on Monod-type equations or application of FBA. Biochemical resource allocation models will allow us to represent the microbial diversity observed in almost all environments and will open up new avenues to interface biogeochemical and ecological questions with recent knowledge obtained from quantitative microbial growth physiology.

## 10. Material and methods

### 10.1. Biochemical resource allocation models

We consider a particular variant of biochemical resource allocation models following the methods and algorithms described in [31,32,35]. A model consists of two types of components: steady-state metabolites and cellular macromolecules (which include catalytic protein complexes and quota components). We assume that the internal metabolites are at a quasi-steady state, i.e. metabolites adjust faster than any changes in the environment. Thus, the concentrations of internal metabolites are not explicitly represented in the model, and the metabolic network is assumed to be balanced at all times. We also neglect dilution by growth of internal metabolites. Catalytic components (including enzymes and ribosomes) are synthesized from the precursors provided by cellular metabolism. The amounts of catalytic components provide an upper bound to the respective rates of the reaction catalysed by the components. The quota components (protein *P*_*Q*_ and remaining biomass *Q*) fulfil no explicit functional role within our model and their synthesis is enforced using fixed quotas (except otherwise noted, the quota protein component *P*_*Q*_ is assumed to be 20% of total protein, the non-protein biomass *Q* is assumed to be 50% of total biomass).

### 10.2. The biochemical resource allocation model of phototrophic growth

The biochemical resource allocation model shown in figure 1 is assembled using the stoichiometry and data described by Faizi *et al.* [26] (with modifications described below). We note that the model of Faizi *et al.* [26] is a nonlinear kinetic ODE model, hence computationally different from the model described here. Growth is facilitated by eight protein complexes: six enzyme and transport complexes, ribosome R and a non-catalysing quota protein component *P*_*Q*_. The enzyme and transporter complexes catalyse the following reactions:

10.1 $$\begin{array}{rl} v_{\mathrm{PSU}}(\text{PSU}):\; & 8\cdot \text{photons}⟶8\cdot e+O_{2}\\ \;v_{TC}(T_{C}):\; & C_{x}+e⟶C_{i}\\ v_{\mathrm{CB}}(\text{CB}):\; & 3\cdot C_{i}+10\cdot e⟶C_{3}\\ v_{\mathrm{MC}}(M_{C}):\; & 2\cdot C_{3}+2\cdot N+35\cdot e⟶\text{AA}\\ v_{\mathrm{TN}}(T_{N}):\; & N_{x}+e⟶N\\ \;\text{ and}\;v_{\mathrm{MQ}}(M_{Q}):\; & C_{3}⟶Q. \end{array}\}$$

Protein translation is described by the equation

10.2 $$\gamma_{P}(R):\;n_{p}\cdot \text{AA}+3n_{p}\cdot e⟶\text{protein},$$

where *n*_p_ denotes the size of the respective protein in amino acids. Protein decay is neglected except for the protein complex PSU that is damaged by light (photoinhibition), such that its abundance follows the equation

10.3 $$\frac{\text{d}[\text{PSU}]}{\text{d}t}=\gamma_{\mathrm{PSU}}-v_{d}-\mu \cdot [\text{PSU}],$$

with

10.4 $$v_{d}:\;\text{PSU}⟶n_{\mathrm{PSU}}\cdot \text{AA}.$$

The dynamics of all other protein complexes are modelled according to equation (4.1).

### 10.3. Capacity constraints of catalytic enzymes

All enzyme-catalysed reactions are constrained by the amount of the respective catalysing enzyme, according to equation (4.3). The constraints for nutrient uptake and light harvesting also depend on the availability of the respective external substrates. In particular, for the uptake of inorganic carbon,

10.5 $$v_{TC}\leq \frac{C_{x}}{K_{C}+C_{x}}\cdot k_{\mathrm{cat},T_{C}}\cdot T_{C},$$

for uptake of extracellular nitrogen,

10.6 $$v_{TN}\leq \frac{N_{x}}{K_{N}+N_{x}}\cdot k_{\mathrm{cat},T_{N}}\cdot T_{N},$$

and for light harvesting and photosynthesis

10.7 $$\begin{array}{rl} & v_{\mathrm{PSU}}\leq \frac{k_{\mathrm{cat},\mathrm{PSU}}\cdot \sigma I}{\sigma I+k_{\mathrm{cat},\mathrm{PSU}}+k_{d}\cdot \sigma I}\cdot \text{PSU}\\ \;\text{and}\; & v_{d}=\frac{k_{d}{\left(\sigma I\right)}^{2}}{\sigma I+k_{\mathrm{cat},\mathrm{PSU}}+k_{d}\cdot \sigma I}\cdot \text{PSU}. \end{array}$$

The equations for light harvesting, photosynthesis and photodamage, are derived from a simple two-state model of photosynthesis, see [26] for details. We note that, different from most other constraints, the rate of photodamage is subject to an equality constraint. That is, the reaction is enforced to proceed according to the specified rate. Along similar lines, also other external parameters or chemical compounds may modulate or enforce specific reactions if required for a specific model.

The constraints on the ribosomal capacity are

10.8 $$\sum_{p}\gamma_{p}\cdot n_{p}\leq \gamma^{\max}\cdot R,$$

where *n*_p_ denotes the protein size (in amino acids per molecule), *γ*_p_ its translation rate and *γ*^max^ denotes the maximal translation rate of ribosomes. We note that all capacity constraints are implemented as linear constraints.

### 10.4. Solving the resource allocation model as a LP

For any given set of external parameters *C*_*x*_, *N*_*x*_, *I* and specific growth rate *μ*, the model implemented as a LP(*μ*). The LP problem is described by three matrices ***N***, ***W*_≤_**, and ***W_=_***, as well as the vector of reaction rates ***v*** = (*v*_*i*_, *γ*_*k*_)^*T*^ (combining metabolic and translation rates), the vector ***P*** of (the concentrations of) macromolecules,

10.9 $$P={\left(R,T_{N},T_{C},\text{PSU},M_{C},M_{Q},\text{CB},P_{Q},Q\right)}^{T},$$

and a vector ***ω*** that describes the sizes of the macromolecules (in units of amino acids per molecule). For each macromolecule, we define a lower bound that ensures the synthesis of quota components,

10.10 $$P^{lb}={\left(R^{lb},T_{N}^{lb},T_{C}^{lb},{\text{PSU}}^{lb},M_{C}^{lb},M_{Q}^{lb},{\text{CB}}^{lb},P_{Q}^{lb},Q^{lb}\right)}^{T}.$$

The matrix ***N*** describes the stoichiometry of the system, including metabolic and translation reactions, as well as the stoichiometry of the decay of the PSU as a result of photodamage (*v*_*d*_). The matrices ***W*_≤_** and ***W_=_*** describe the relationship between reaction fluxes and ***P***. The entries of both matrices may depend on the external parameters *C*_*x*_, *N*_*x*_ and *I*.

The full set of constraints is

10.11 $$\begin{array}{rl} & N\cdot v=\mu \cdot \left[\begin{array}{c} 0\\ P \end{array}\right], \end{array}$$

10.12 v≥0,

10.13 $$W_{\leq }\cdot v\leq P,$$

10.14 $$W_{=}\cdot v=P,$$

10.15 $$P\geq P^{lb}$$

10.16 andω⋅P=d.

Constraint (10.11) enforces mass-balance at steady-state, including the growth-induced dilution of macromolecules (with dilution of metabolites neglected). Constraint (10.13) describes the (linear) enzymatic capacity constraints with the concentration of macromolecules as upper bounds. The matrix ***W***_≤_ is largely diagonal, except for the constraints on the translation rate. Constraint (10.14) describes enforced reactions that are proportional to the abundance of a macromolecule (such as photoinhibition). Constraint (10.15) provides a lower bound for the abundance of each macromolecules (zero except for the quota components). Constraint (10.12) ensures positive fluxes in the LP problem. Constraint (10.16) enforces a constant cell density, the parameter *d* denotes the cell density (in units of amino acids per cell).

The above described LP is solved as a feasibility problem for a given *μ*. To obtain a solution for the maximal specific growth rate in a given environment, the global optimum of *μ* is found using bisection as described in [31].

### 10.5. Model parametrization

A complete list of model parameters is provided in table 2. Parametrization follows the data used in Faizi *et al.* [26]. The size of macromolecules is estimated using the size of an average enzyme times the approximate number of steps used in the pathway. The size of the protein complex *P*_Q_ and the biomass component *Q* is arbitrary. Turnover rates are chosen according to average values described in [50]. The description of the photosystem is adopted from Faizi *et al.* [26], with *σ* denoting the effective absorption cross-section per photosystems, and *k*_d_ the rate of photodamage.

To simulate the growth on two alternative sources of external nitrogen, we used the set of additional parameters given in table 3.

**Table 3.** Specific parameters used to model growth on two alternative sources of external nitrogen. The remaining parameters are same as described in table 2.

symbol	definition	(*T*_N_)-strain	(*T*_Y_)-strain	(*T*_N_ + *T*_Y_)-strain
*e*_N_	energy units per uptake reaction *T*_N_	1	—	1
*e*_Y_	energy units per uptake reaction *T*_Y_	—	2	2
$k_{\mathrm{cat},T_{Y}}$	catalytic activity of *T*_Y_ (s^−1^)	—	30	30
$k_{\mathrm{cat},T_{N}}$	catalytic activity of *T*_N_ (s^−1^)	50	—	50
$n_{T_{N}}$	size of *T*_N_ (aa molec^−1^)	10 000	—	10 000
$n_{T_{Y}}$	size of *T*_Y_ (aa molec^−1^)	—	20 000	20 000
*Q*	relative abundance of *Q* w.r.t. biomass	0.5	0.5	0.6
*P*_*Q*_	relative abundance of *P*_Q_ w.r.t. total proteome	0.2	0.2	0.22

### 10.6. The computational modelling framework

A computational model is developed using Python as a programming language. The framework uses functionality from the following packages: numpy [70], scipy [71], matplotlib [72], pandas [73], sundials [74] and Gurobi [75]. In particular, we use Gurobi for solving the LP-based optimization and CVODE integrator from the sundials package to solve the system of ODEs. The version of the modelling framework used to produce the results presented in this manuscript is publicly available with instructions to install and run simulations at (https://github.com/surajsept/cyanoRBA).

## Data accessibility

The code used in the simulations is available at https://github.com/surajsept/cyanoRBA.
